# Research progress on risk factors of delirium in burn patients: A narrative review

**DOI:** 10.3389/fpsyt.2022.989218

**Published:** 2022-11-02

**Authors:** Yujie Ren, Yu Zhang, Jinhua Luo, Wenqiang Liao, Xing Cheng, Jianhua Zhan

**Affiliations:** ^1^Medical Center of Burn Plastic and Wound Repair, The First Affiliated Hospital of Nanchang University, Nanchang, Jiangxi, China; ^2^Medical Innovation Center, The First Affiliated Hospital of Nanchang University, Nanchang, China

**Keywords:** burn injury, delirium, risk factors, mental disorders, diagnosis

## Abstract

Delirium, an acute brain dysfunction, is a common and serious complication in burn patients. The occurrence of delirium increases the difficulty of patient treatment, is associated with various adverse outcomes, and increases the burden on the patient’s family. Many scholars have studied the factors that cause delirium, but the causes, pathogenesis, and treatment of delirium in burn patients have not been fully revealed. There is no effective pharmacological treatment for delirium, but active preventive measures can effectively reduce the incidence of delirium in burn patients. Therefore, it is necessary to study the relevant factors affecting the occurrence of delirium in burn patients. This study was conducted on December 20, 2021 by searching the PubMed database for a narrative review of published studies. The search strategy included keywords related to “burns,” “delirium,” and “risk factors.” We reviewed the characteristics of delirium occurrence in burn patients and various delirium assessment tools, and summarized the risk factors for the development of delirium in burn patients in terms of personal, clinical, and environmental factors, and we found that although many risk factors act on the development of delirium in burn patients, some of them, such as clinical and environmental factors, are modifiable, suggesting that we can estimate the exposure of burn patients to risk factors by assessing their likelihood of delirium occurring and to make targeted interventions that provide a theoretical basis for the prevention and treatment of burn delirium.

## Introduction

Burn injuries have a high incidence in daily life (1), making burns one of the four major traumas on the planet. Burns, especially severe burn injuries, can cause multiple organs and multiple system damage in individuals, so burn patients usually face a complicated treatment that includes wound dressing changes, anti-infective, anti-shock, sedation, analgesia management, and even one or more operations (2, 3). During the painful and lengthy treatment procedure, many burn patients are susceptible to various mental disorders (4). Burns and mental disorders have a complicated relationship; roughly 61% of adult burn patients with an average burn area of 9% of total body surface area (TBSA) experienced mental disorders, particularly delirium (4). Delirium is acute organic brain dysfunction, a clinical syndrome caused by multiple factors and characterized by coexisting attention, awareness, and cognition disturbances (5). Delirium pathogenesis is not yet fully elucidated but has proposed several hypotheses. The popularly accepted doctrines include the neurotransmitter imbalance hypothesis, the stress response hypothesis, the inflammatory hypothesis, the melatonin dysregulation hypothesis, and the brain metabolic imbalance hypothesis (6–8). Recently, there has been a progressive interest in delirium as it can cause adverse results such as prolonged hospital stay (9, 10), increased mortality (11, 12), greater hospitalization costs (13, 14), and even cognitive decline for a long time after discharge (15). However, little attention has been paid to discussing delirium caused by burns, and there is no clear efficacy of pharmacological treatment of delirium (16, 17). Though, interventions can effectively reduce the risk of delirium in high-risk patients (18) and are more effective in preventing delirium (5). This article aims to provide a reference for preventing and treating burn-induced delirium by reviewing the research progress on risk factors for burn-induced delirium.

## Methods

**FIGURE 1:**
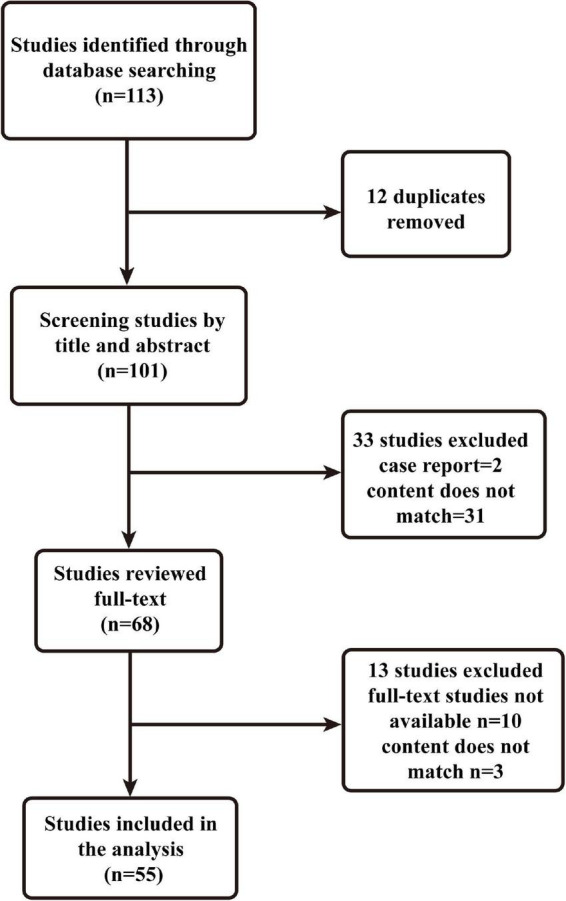
Flowchart of the selection process.

A narrative review is a traditional method of collecting a wide range of literature around a topic, analyzing and synthesizing its content, and ultimately reporting on relevant theories, ideas and an overview of developments (19). This manuscript provides a narrative review of risk factors for the development of delirium in patients with burns. Two authors developed a search plan (Table 1). On December 20, 2021, we searched and analyzed all literature related to the keywords “burns,” “delirium,” and “risk factors” using the PubMed database. We did not set a time limit for publication. Inclusion criteria were mate analysis, narrative reviews, systematic reviews, clinical trials (retrospective and prospective studies), exclusion criteria: case reports, basic studies, and animal experiments. The two authors performed the inclusion and exclusion of literature (Figure 1), and corresponding authors were consulted when there was disagreement. A total of 55 papers were eventually included.

**TABLE 1:** Search literature summary.

Items	Details
Database	PubMed database
Search time	December 20, 2021
Keywords	“burn,” “delirium,” and “risk factors”
Timeline	1950-present
Inclusion criteria	Mate analysis, narrative reviews, systematic reviews, clinical trials (retrospective and prospective studies)
Exclusion criteria	Case reports, basic studies, and animal experiments
Sample size	55
Selection process	Two authors developed a search plan and performed the inclusion and exclusion of literature, consulting with a corresponding author when there was disagreement

### Epidemiology and diagnosis

The prevalence of delirium in burn patients ranges from about 13 to 80% (4, 12, 20–23). The rates appear in a treatment setting. In Burn Intensive Care Unit (BICU), the rate is around eight times higher than in the burn general ward (20), and delirium occurs in roughly 77% of burn patients receiving mechanical ventilation (MV) in BICU (22). Delirium is classified into three types (6): hypoactive (characterized primarily by apathy, drowsiness, reduced alertness, and motor activity), hyperactive (characterized primarily by agitation, hallucinations, and delusions), and mixed (both of these manifestations). Because of the specificity of its manifestations, the most common subtype of delirium is hypoactive delirium (24, 25), in which the condition is easily overlooked and usually requires assessment tools. Both the *Diagnostic and Statistical Manual of Mental Disorders* (DSM) and the *International Classification of Diseases* (ICD) have contributed to the diagnosis of delirium. However, the DSM is mostly used as a diagnostic criterion in research and clinical work, probably because the ICD is more restrictive and less accurate in the diagnosis of delirium (26, 27). As a result, the more inclusive (28) *Manual of Mental Disorders–Fifth Edition* (DSM-5) (29–32) has been used as the standard for research and clinical work. More than 50 delirium assessment tools have been developed for use in various clinical settings (33), such as the Confusion Assessment Method (CAM) (34), the Ambiguous Assessment of Consciousness in the Intensive Care Unit (CAM-ICU) (35), the Intensive Care Delirium Screening Checklist (ICDSC) (36), the Nursing Delirium Screening Scale (Nu-DESC) (37). However, inaccurate delirium documentation resulting from some medical staff questioning and misusing delirium assessment tools has resulted in some delirious patients not being taken seriously (38), implying that burn centers need to recognize the importance of early diagnosis of delirium and routinely screen for delirium assessment during the treatment of burn patients to avoid exacerbating the condition of under-diagnosed patients. In recent years, scholars have worked to find more objective indicators for the diagnosis of delirium, such as biomarkers (39) and Electroencephalography (EEG) (40). Several biomarkers (41) have been used to investigate the feasibility of diagnosing delirium, especially inflammatory markers such as C-reactive protein (42) and neutrophil-lymphocyte ratio (NLR) (43), although many biomarkers have been studied, they have been used mainly in experimental studies and have not been used clinically. When delirium occurs in patients, neurophysiological abnormalities occur, mainly in the form of slowed resting-state EEG rhythms, increased theta and delta frequencies, and decreased alpha power (40, 44). When the delirium improves, the EEG returns to normal (44). Therefore, EEG can diagnose whether a patient is experiencing delirium by identifying neurophysiological abnormalities in the patient. However, due to the lack of high-quality prospective experiments, EEG is currently used only to identify delirium and non-convulsive persistent epilepsy or psychiatric disorders in clinical practice (5, 18).

### Risk factors

Delirium is a complex disease caused by several factors, the mechanism of which has not been fully elucidated yet. To reduce the occurrence of delirium in burn patients, we should start to prevent delirium from its risk factors. The risk factors leading to delirium in burn patients (Table 2) are summarized as follows.

**TABLE 2:** Risk factors for delirium in burn patient’s summary.

Items	Details
Personal factors	Advanced age, dementia, low educational attainment, diabetes, hypertension, heart disease, history of alcohol use, history of smoking
Clinical factors	Severity of burn injury, benzodiazepines, opioids, anticholinergic drugs, surgical treatment, blood transfusion, pain, infection, hypoxemia, MV, dysglycemia, electrolyte disorders
Environmental factors	Sleep deprivation, circadian rhythm disorders, physical restraint, absence of family companionship

MV, mechanical ventilation.

#### Personal factors

##### Advanced age

Burns can occur at any age, and advanced age is one of the risk factors for developing delirium in burn patients (12, 45–47). The incidence of delirium is significantly higher in burn patients over 65 (8). Abdelrahman et al. argued that age greater than 75 years is an independent risk factor for developing delirium in burn patients (20). This may be because elderly patients have more underlying diseases and a decrease in all physiological functions with increasing age, resulting in the decreased metabolic function of brain cells, decreased cerebral blood flow (48), decreased vascular density (49), and progressive degeneration of brain tissue (50, 51). Moreover, older patients have reduced neurotransmitter synthesis (8), increasing the delirium risk.

##### Education attainment

The education level is considered to be a proxy for the cognitive reserve (52). Patients with low educational attainment in multiple clinical settings are now more prone to delirium (53–55), consistent with the previous findings (52). In a multicenter epidemiological study of burn patients (56), it was found that elderly burn patients tend to live in rural areas and have low educational attainment. They may lack adequate knowledge of burn prevention and increase the risk of burn injury, and such patients may delay seeking medical attention after a burn injury for various reasons leading to a serious condition. Moreover, some low-education groups may be engaged in manual work, and their financial capacity is not optimistic. They not only have to bear the physical pain of the burn injury but also the additional costs of treatment and the psychological stress of later functional rehabilitation and whether they can return to society. People under prolonged stress may impair brain function, leading to psychiatric disorders (57) and an increased risk of delirium.

##### Pre-existing diseases

Pre-existing diseases in burn patients are also associated with the development of delirium. Evidence supports diabetes (58), hypertension (59–62), and heart disease (6, 54) as risk factors for the development of delirium in burn patients. Long-term hypertension impairs memory and attention (63), and the adverse effects on blood vessels lead to impaired vascular regulation (64). Severe burn patients are prone to hypovolemic shock. Although provided fluid resuscitation during treatment, burn patients with combined hypertension tend to have cerebral ischemia and hypoxia due to impaired vascular pressure regulation, which affects brain energy metabolism thereby increasing the risk of delirium (65). Diabetes also impairs vascular regulation and causes cognitive decline (66) in patients. Patients with hypertension, diabetes and heart disease are prone to cerebral ischemia and hypoxia after severe burns, affecting central nervous system function and brain energy metabolism (65). All these factors can combine to contribute to the occurrence of delirium.

Both dementia and delirium exhibit cognitive impairment, and burn patients with comorbid dementia are more likely to develop delirium (45, 59, 67–69). In an examination by Holmes, the hospitalization risk for burn injuries in dementia patients was as high as 60% (23, 67). There is a subtle association between the two: the elderly burn patients with dementia are more likely to develop delirium (70), and delirium development can further exacerbate dementia (71), thereby increasing delirium risk (6, 72). Patients who develop delirium have increased markers of axonal injury (73, 74) and develop markers of synaptic damage (75), persistent cognitive deficits, and hippocampal atrophy (76). These suggest that delirium may exacerbate dementia in patients by causing new brain damage. Because delirium and dementia have similar clinical manifestations, it is critical to be aware of delirium when a burn injury occurs in a patient with dementia and actively assess the correct diagnosis to avoid misdiagnosis and aggravation of the patient’s condition.

##### Lifestyle

Some burn patients with alcohol use (62) and smoking (77) are more likely to develop delirium. This result could be explained in various ways. Long-term heavy liquor consumption affects patients’ cognitive abilities (59, 60). Moreover, it increases the probability of alcohol withdrawal syndrome after hospital admission due to sudden interruption of drinking, which may result in withdrawal episodes and delirium tremens (78). However, further research is needed to determine whether there is a link between the amount of alcohol consumed by burn patients and the severity of delirium. Patients who are smoking are asked to quit upon admission and acute nicotine withdrawal can drive the development of delirium, especially hyperactive delirium (79). Smoking can also adversely affect patients’ lung function while causing cognitive decline (80). When such patients have inhalation injury, it is easy to aggravate the lung injury and lead to poor respiratory function, which may require MV to improve breathing, promoting delirium development (81).

#### Clinical factors

##### Medications

Burns are multi-organ and multi-system damage and require comprehensive treatment. Burn patients are faced with complex sedation and analgesic management. Benzodiazepines are the most used sedative drugs for burns (82). One of the reasons benzodiazepines are the most commonly used sedative drugs for burns is that they are probably safer for patients with hemodynamic instability shock-phase burns (83). Benzodiazepines are independent factors in the development of delirium, particularly lorazepam, midazolam (7, 22), and benzodiazepines can also influence the duration of delirium in patients (84). γ-aminobutyric acid (GABA) may increase delirium prevalence by enhancing central nervous system (CNS) depression and destabilizing the sleep cycle (85). Benzodiazepines, on the other hand, enhance GABA’s inhibitory effect by binding to its A receptors, disrupting the effective connections between brain regions that cause a resting state of consciousness and contributing to the development of delirium (85). This finding suggests that benzodiazepines should be used cautiously in treating burn patients and that adequate analgesia can reduce the need for sedative drugs.

Analgesic treatment of burn patients is a very effective therapeutic measure to reduce or eliminate stress and pathophysiological damage to the body in response to nociceptive stimuli. Opioids are frequently used to provide analgesia. Patients who receive intravenous opioids are more likely to develop delirium (86, 87). This finding could be explained by the fact that opioids can increase glutamate and dopamine activity while altering acetylcholine and GABA (23). However, there is a rather unexpected outcome that using low doses of opioids reduces the prevalence of delirium when patients present with acute severe pain (22, 87–89), probably because small doses result in lower drug levels in the blood and achieve the desired analgesic effect.

The cholinergic system is an important neurotransmitter regulatory system primarily associated with cognition and attention (8). Many hypotheses for the pathogenesis of delirium have been proposed. The most common neurotransmitter change in the neurotransmitter imbalance hypothesis is a decrease in the availability of acetylcholine (8). Burn patients taking anticholinergic medications are much more likely to develop delirium than those who are not (12, 90). When acetylcholine levels are insufficient in burn patients, cognition and attention are affected; thus, delirium occurs (91). However, not all drugs with anticholinergic effects can increase the incidence of delirium. This is evident in the case that morphine has a slight anticholinergic effect; when used appropriately, it reduces the risk of delirium development, which may also be related to its analgesic effect (22).

##### Surgery

The effect of surgery on burn delirium is complex and multifactorial. Burn patients, especially those with severe burns, face general anesthetic procedures during the treatment, and the number of procedures (20) and the excessive duration of the procedure (92) influence the occurrence of delirium. This may be due to the more and longer general anesthesia procedure, the more sedative and analgesic drugs used during the procedure, and the more toxic substances produced by metabolic disturbances, which affect the development of combined delirium in burn patients (22, 86, 87). Furthermore, the surgical approach in burns is significantly different from minimally invasive surgery in other disciplines, which tends to cause massive openings of vascular beds. With the increase of intraoperative bleeding and exudation, it is easy to have intraoperative hypotension causing insufficient brain tissue perfusion leading to ischemia and hypoxia, thus increasing the risk of delirium in burn patients (92).

##### Other treatments

Patients with head and facial burns often have combined inhalation injuries. They are often associated with respiratory dysfunction, leading to hypoxemia (93), which ultimately leads to delirium by causing decreased acetylcholine production (8) and cerebral ischemia and hypoxia. MV is often used for hypoxemia that cannot be resolved by oxygenation, and the incidence of delirium in burn patients on MV can be as high as 77% (22, 81, 94). Far too little attention has been paid to the mechanism by which MV increases the risk of delirium is unclear and may be related to the fact that MV can rapidly lead to brain damage expressed as delirium (81).

Patients who experience burns have increased vascular permeability and more exudate resulting in reduced blood volume (95), often resorting to blood transfusion to combat hypovolemic shock. In addition to the above, intraoperative blood transfusion is usually an option for burn patients due to the high intraoperative bleeding and exudation associated with their surgical approach. However, little research has been conducted on the mechanism; blood transfusion may affect neuroinflammation and oxidative stress (96), thereby driving delirium development (96, 97).

##### Severity of burn

The burn severity is determined by its surface area and depth. According to Abdelrahman et al. (20), burn patients with more than 30% TBSA are more likely to develop delirium (21), who discovered that the burn surface area of patients in the delirium group was three times greater than that of patients in the non-delirium group, ranging from approximately 67 to 77% (20, 21). Whereas, the prevalence of delirium is less than 10% in patients with less than 10% TBSA (20), the rate can be up to 27% in patients with 10–30% TBSA. The reasons for this are manifold. The management of sedation and analgesia in patients with large burns is more problematic, and more sedative and analgesic drugs are used. Patients with severe burns are also exposed to multiple surgical procedures, and the procedure’s length (98), intraoperative anesthetic drugs (87), and postoperative pain (60) can influence the onset of delirium. Moreover, they require a blood transfusion (97) to combat hypovolemic shock. This group of patients is also at greater risk of infection (20). For example, some patients with severe burns require the opening of venous channels for rehydration and anti-shock. The choice of deep venous catheterization points and the length of placement time will directly impact the occurrence of sepsis in burn patients. Together, these factors contribute to the onset of delirium. However, a systematic understanding of how the depth of burns contributes to delirium is still lacking.

##### Pain and infection

Pain is unavoidable in burn patients’ treatment. Operative pain (wound cleaning, dressing changes, catheter care, etc.) is common in burn patients. Pain is a risk factor for burn-induced delirium (8, 60, 99). This is because acute pain increases oxygen consumption due to the stress reaction, affects brain cells’ metabolic function, and lowers the threshold for delirium onset by inducing catecholamine release (57). On the other hand, pain can affect sleep in burn patients leading to sleep-wake cycle disruption and sleep deprivation, causing nociceptive overreaction to increase delirium risk (100).

Infection is another risk for burn patients. When the skin, the body’s natural barrier against external bacterial invasion, is damaged by burning, necrotic skin tissue becomes a suitable culture medium for microorganisms. Moreover, traumatic stress, shock, and suppression of intrinsic immune function occur simultaneously or sequentially, making the wounds highly susceptible to infection and even sepsis. Burn patients, particularly those with extensive burns, are susceptible to infection due to trauma, intravenous placement, respiratory and intestinal tracts, or various medical factors. Abdelrahman et al. (20) point out a correlation between infectious inflammation and delirium, possibly because the infection may be due to inflammation that leads to the release of inflammatory mediators from macrophages and cerebrovascular endothelial cells, which affect neurological function (8). Burn patients with infections usually present with fever; in severe cases, they may develop persistent hyperthermia. Under the influence of persistent high fever, the patient’s level of consciousness decreases, and they are prone to delirium marked by agitation and hallucinations (101).

##### Electrolyte disturbance and glucose abnormality

Electrolyte disturbances and glucose abnormality also promote delirium (8, 102). Under normal circumstances, brain function is dependent on glucose oxidation. The occurrence of delirium in pediatric burn patients is closely related to hyponatremia and hypoglycemia (93), which affects brain function and acetylcholine synthesis (103). Stress hyperglycemia is associated with extensive burns (104), which causes delirium by affecting intra- and extracellular osmolarity and promoting the release of inflammatory factors (105).

#### Environmental factors

Because burn patients have high requirements for ward environment and sterility, the center mandates that patients’ families enter the burn general ward for regular visits. However, after COVID-19, the burn general ward, like the BICU, was also closed to family members. Meanwhile, Patients who are continuously alone in an unfamiliar environment after burn injury and lack communication and encouragement from family members are prone to mood depression. Patients with severe burns are mostly in BICU, surrounded by more resuscitation patients. Patients in the adjacent wards are susceptible to great stress and panic in this process, increasing delirium risk (106).

Furthermore, delirium can be caused by disruptions in circadian rhythms, changes in natural light exposure, and low melatonin levels (100, 107). Moreover, during hospitalization, the sound of machines, the operation of medical staff, the lighting of the ward, and the activities of other patients in the same ward can disrupt the patient’s sleep, causing disturbances in the physiological sleep architecture and, thus, delirium (108). The use of physical restraint for some burn patients (107, 109), particularly pediatrics, prevents serious consequences from falling out of bed. Pediatric patients subjected to physical restraint are more likely to develop delirium (110), and physical restraint exacerbates delirium (111). This may be related to physical restraint increasing the patient’s fear and anxiety.

## Conclusion

Delirium is a frequent and severe complication of burn injury, predisposing patients to increased length of hospital stay (10), mortality (12), and medical and social costs (13). It is a multifactorial cause of acute brain dysfunction that develops rapidly and poses a potential threat to every burn patient. Understanding the mechanisms of burn-induced delirium, modifying the risk factors present in burn patients, and improving treatment protocols will facilitate the comprehensive treatment of burn patients. The delirium pathogenesis has not been fully uncovered, and there are significant challenges to its screening, prevention, and treatment. The clinical challenges in managing delirium in burn patients lie, on the one hand, in the lack of awareness of delirium among medical staff and the failure to standardize the learning and use of delirium assessment tools (38). On the flip side, fewer early prediction models for delirium in burn patients exist. Although many predictive models are based on other diseases combined with delirium, these models are mostly internally validated and lack external validation (112). Furthermore, there is also a lack of validation for applicability to burn patients. Therefore, developing and validating an early prediction model of delirium belonging to burn patients is one of our next priorities. This manuscript summarizes the risk factors for complications of delirium in burn patients as personal factors (advanced age, dementia, low educational attainment, diabetes, hypertension, heart disease, history of alcohol use, and smoking history), clinical factors (severity of burn injury, benzodiazepines, opioids, anticholinergic drugs, surgical treatment, blood transfusion, pain, infection, hypoxemia, MV, dysglycemia, electrolyte disorders) and environmental factors (sleep deprivation, circadian rhythm disorders, physical restraint, absence of family companionship) in three areas. The management of delirium in burn patients requires a joint effort by doctors and nurses. Delirium is primarily prevented and treated through non-pharmacologic approaches (18), and pharmacological prevention and treatment (16, 17) is not a first-line option. Some of the risk factors mentioned above, such as advanced age and low educational attainment, cannot be changed. We mainly intervene in the modifiable risk factors. Upon admission, patients are strictly managed for pre-existing conditions, blood glucose and blood pressure are controlled, the cardiopulmonary function is assessed, and the underlying disease is managed in a joint multidisciplinary approach. Avoid or use cautious medications that may cause delirium in patients, such as benzodiazepines (22), opioids (87), and anticholinergics (12). For the management of sedation and analgesia in burn patients, safer medications are used, routine pain assessment is conducted, excessive sedation is avoided, and gradual and slow withdrawal to avoid triggering withdrawal reactions (113). During treatment, the amount of blood transfused to the burned patient is strictly controlled according to the actual situation of rehydration, and electrolyte disorders and hypoxemia are also actively corrected. Opt for early surgery while avoiding unnecessary catheter placement, performing microbiological cultures, and selecting drugs for the infection to effectively control it. Use physical restraints with caution, and for patients already in physical restraints, conduct regular assessments to avoid using them for too long. Optimize the patient’s ward environment. As burn patients live in isolation wards, family members cannot visit them and can use the telephone to communicate with their families. Moreover, they are also encouraged to place items familiar to the patient next to their bed. Reduce the sound of staff or equipment in the ward and reduce the number of medical or nursing procedures at night. Avoid the use of lighting at night and provide patients with eye or ear plugs if necessary to maintain a normal sleep-wake cycle. When delirium patients are ineffective with non-pharmacological approaches and are in a state of severe agitation, pharmacologic treatment such as anti-psychotics can be considered (18). Complications of delirium such as falls (114) should also be actively prevented during treatment, and recovery should be monitored. The limitation of our study is that at this stage there are more studies on patients with delirium in the intensive care unit and other surgical settings and less exploration of burn injury combined with delirium, hence the length of the studies we included. And there are some factors that influence the severity of burns that have not been studied to explore whether they are related to the development of delirium, such as the depth of burns. Also, due to resource constraints, we mostly used English studies and Chinese studies, and did not review articles in other languages. At the same time our study is a narrative review, which is somewhat different from a systematic review. A systematic review is a critical evaluation of issues related to a topic using specific and reproducible strategies and aims to reduce bias (115). However, narrative reviews suffer from shortcomings such as poor reproducibility (116) and bias (19, 117). As a future work, we will conduct a systematic review of risk factors and mechanisms of delirium occurrence in burn patients and explore the influence of factors such as burn depth and area on the incidence of delirium in patients, as well as conduct a study of delirium prediction models. In addition, we believe that this review highlights the importance of the fact that adequate recognition and effective control of risk factors for delirium and the implementation of early screening will be beneficial in reducing the incidence of delirium and provide a corresponding reference value for the treatment and prevention of delirium. It also provides clinical ideas for establishing early predictive models, finding effective biomarkers, and exploring more effective therapeutic drugs and methods, among other measures. It enables patients to improve psychologically and physically and to integrate into society early.

## Author contributions

Y-JR and YZ: conceptualization and writing—original draft. J-HL and W-QL: visualization. XC: writing—review and editing. J-HZ: writing—review and editing and funding acquisition. All authors contributed to the article and approved the submitted version.
